# Acute Auditory Stimulation with Different Styles of Music Influences Cardiac Autonomic Regulation in Men

**Published:** 2014-09-01

**Authors:** Sheila Ap. F. da Silva, Heraldo L. Guida, Ana Marcia dos Santos Antonio, Luiz Carlos de Abreu, Carlos B. M. Monteiro, Celso Ferreira, Vivian F. Ribeiro, Viviani Barnabe, Sidney B. Silva, Fernando L. A. Fonseca, Fernando Adami, Marcio Petenusso, Rodrigo D. Raimundo, Vitor E. Valenti

**Affiliations:** 1 Center for the Study of Nervous System (CESNA), Department of Speech Therapy, Faculty of Sciences, UNESP, Marilia, SP, Brazil; 2 Postgraduate Program in Physiotherapy, Faculty of Technology Sciences, UNESP, Presidente Prudente, SP, Brazil; 3 Department of Morphology and Physiology, Faculty of Medicine of ABC, Santo Andre, SP, Brazil; 4 Division of Cardiology, Department of Medicine, UNIFESP, Sao Paulo, SP, Brazil; 5 Department of Environmental Health, Harvard Medical School of Public Health, Boston, MA, USA

**Keywords:** Autonomic Nervous System, Cardiovascular System, Music, Auditory Stimulation

## Abstract

**Background:**

No clear evidence is available in the literature regarding the acute effect of different styles of music on cardiac autonomic control.

**Objectives:**

The present study aimed to evaluate the acute effects of classical baroque and heavy metal musical auditory stimulation on Heart Rate Variability (HRV) in healthy men.

**Patients and Methods:**

In this study, HRV was analyzed regarding time (SDNN, RMSSD, NN50, and pNN50) and frequency domain (LF, HF, and LF / HF) in 12 healthy men. HRV was recorded at seated rest for 10 minutes. Subsequently, the participants were exposed to classical baroque or heavy metal music for five minutes through an earphone at seated rest. After exposure to the first song, they remained at rest for five minutes and they were again exposed to classical baroque or heavy metal music. The music sequence was random for each individual. Standard statistical methods were used for calculation of means and standard deviations. Besides, ANOVA and Friedman test were used for parametric and non-parametric distributions, respectively.

**Results:**

While listening to heavy metal music, SDNN was reduced compared to the baseline (P = 0.023). In addition, the LF index (ms^2^ and nu) was reduced during exposure to both heavy metal and classical baroque musical auditory stimulation compared to the control condition (P = 0.010 and P = 0.048, respectively). However, the HF index (ms^2^) was reduced only during auditory stimulation with music heavy metal (P = 0.01). The LF/HF ratio on the other hand decreased during auditory stimulation with classical baroque music (P = 0.019).

**Conclusions:**

Acute auditory stimulation with the selected heavy metal musical auditory stimulation decreased the sympathetic and parasympathetic modulation on the heart, while exposure to a selected classical baroque music reduced sympathetic regulation on the heart.

## 1. Background

Heart Rate Variability (HRV) is a non-invasive method for investigation of Autonomic Nervous System (ANS). This method analyzes the oscillations of the intervals between consecutive heartbeats (RR intervals) and is conventionally accepted to describe the fluctuations in RR intervals which are indicated to influence the sinusal node (1).

Up to now, a large number of studies on music therapy related to HRV have investigated the subjects under treatment awaiting surgical procedures, or immediately after surgery. However, no studies have focused on acute physiological responses to baroque and heavy metal music on HRV.

Baroque music auditory stimulation was reported by Bernardi et al. to have positive effects on the cardiovascular system (2). These researchers investigated the effects of orchestra and music with vocals on heart rate, respiratory rate, blood pressure, and middle cerebral artery flow. Music was also noted to reduce the activity of the sympathetic nervous system (2).

Conversely, heavy metal music was indicated to cause negative effects related to stress response (3) and increase sympathetic activity. Baroque music, on the other hand, reduced the sympathetic nervous system activity but increased parasympathetic activity (3).

The positive effects of musical auditory stimulation have been reported in long run (4). However, no previous studies have investigated the short-term effects of classical baroque and heavy metal music on HRV. The knowledge of physiological responses induced by music exposure is important to develop future therapies in order to prevent the development of cardiovascular disorders.

## 2. Objectives

Therefore, the present study was undertaken to analyze the acute effects of excitatory heavy metal and classical baroque musical auditory stimulation on HRV in healthy males.

## 3. Materials and Methods

### 3.1. Study Population

This study was conducted on 12 healthy males (Power analysis provided a minimal number of 10 subjects) aging between 18 and 30 years who had referred to our Institution. All the volunteers were informed about the procedures and objectives of the study and signed written informed consents. Besides, all the study procedures were approved by the Ethics Committee of the Faculty of Sciences of the Universidade Estadual Paulista, Campus of Marilia (Protocol No. CEP-2011-382) and followed the resolution 196/96 National Health 10/10/1996.

### 3.2. Exclusion Criteria

The exclusion criteria of the study were having a history of cardiopulmonary and auditory disorders, suffering from neurological and other impairments that did not allow the subjects to perform the procedures, and being under treatment with drugs that influence cardiac autonomic regulation. The subjects with previous experience with musical instruments and classical ballet music as well as those who enjoyed heavy metal and baroque music styles were also excluded from the analysis because these types of music have been suggested to affect the cardiovascular responses (5).

### 3.3. Initial Evaluation

Before the experimental procedure, the volunteers’ age, gender, weight, height, and Body Mass Index (BMI) were identified. Anthropometric measurements were obtained according to Lohman (6). The participants’ weight was measured by using a digital scale (W 200/5, Welmy, Brazil) with the accuracy of 0.1 kg. In addition, their height was determined by using a stadiometer (ES 2020, Sanny, Brazil) with accuracy of 0.1 cm and 2.20 m of extension. Finally, BMI was calculated using the following formula: weight (kg)/height ^2^ (m).

### 3.4. Measurement of the Auditory Stimulation

Equivalent sound levels were measured in a soundproof room using an SV 102 audiodosimeter (Svantek, Poland). It was programmed the measurement in the "A" weighting circuit; slow response.

The measurements were made during a session lasting for four minutes and 50 seconds for the classical baroque music and five minutes and 15 seconds for the excitatory heavy metal music. In doing so, we used the insert type microphone (MIRE - Microphone in real ear) which was placed inside the subjects’ auditory canal just below the microphone connected to the personal stereo.

Before each measurement, the microphones were calibrated with the calibrator acoustic CR: 514 model (Cirrus Research plc). We used in the analysis was the Leq (A), which is defined as the equivalent sound pressure level and corresponds to the constant sound level in the same time interval. It contains the same total energy of the sound. The frequency of the spectrum of the sound stimulation (octave band) was assessed, as well (7-9).

### 3.5. HRV Analysis

After the initial evaluation, the heart monitor strap was placed on each subject’s thorax over the distal third of the sternum. Besides, the HR receiver (Polar RS800CX monitor, Polar Electro OY, Kempele, Finland) was placed on the wrist.

The R-R intervals recorded by the portable HR monitor (with a sampling rate of 1000 Hz) were downloaded to the Polar Precision Performance program (v. 3.0, Polar lectro, Finland). The software enabled the visualization of HR and the extraction of a cardiac period (R-R interval) file in “txt” format. Following digital filtering complemented with manual filtering for the elimination of premature ectopic beats and artifacts, at least 256 R–R intervals were used for data analysis. Only the series with more than 95% sinus rhythm were included in the study (10, 11).

### 3.6. Linear Indices of HRV

To analyze HRV in the frequency domain, the Low Frequency (LF = 0.04 to 0.15 Hz) and High Frequency (HF = 0.15 to 0.40 Hz) spectral components were used in ms^2^ and normalized units (nu), giving a value relative to each spectral component in relation to the total power minus the Very Low Frequency (VLF) components, and the ratio between these components (LF/HF). The spectral analysis was performed using the Fast Fourier Transform algorithm (12).

The analysis of time domain was performed by means of standard deviation of normal-to-normal R-R intervals (SDNN), the percentage of adjacent RR intervals with a difference of duration greater than 50 ms (pNN50), and root-mean square of differences between adjacent normal RR intervals in a time interval (RMSSD) (13, 14).

### 3.7. Experimental Procedures

At first, the participants were located in a room with the temperature between 21 °C and 25 °C and relative humidity between 50% and 60%. They has been instructed not to drink alcohol and caffeine for 24 hours before the evaluation. The data were collected on an individual basis between 8 and 12 AM. All the necessary procedures for data collection were explained on an individual basis and the subjects were instructed to remain at rest and avoid talking during data collection.

The subject remained at seated rest for 10 minutes to record the baseline HRV. Subsequently, the individuals were exposed to each style of music for approximately five minutes at seated rest, since each type of music lasted for around five minutes. HRV was analyzed before exposure to music (control), during baroque music, and during heavy metal music. The sequence of songs was randomized for each individual and there was a 5-minute interval between each music style (Table 1). The subjects remained in seated position all through the experiment. The volunteers were exposed to excitatory heavy metal (Gamma Ray: "Heavy Metal Universe") and classical baroque music (Pachelbel: "Canon" in D Major).

**Table 1.** The Experimental Protocol

Seated Rest	Music (Classical Baroque or Heavy Metal)	Seated Rest	Music (Heavy Metal or Classical Baroque)
10 min	5 min	5 min	5 min

### 3.8. Statistical Analysis

In this study, power test analysis was run, giving a minimum number of 10 volunteers. The sample size was determined using the following formula:

E = zα/2 x Ω/n,

Where the positive z value was considered at the vertical boundary for the area of α/2 in the right tail of the standard normal distribution, zα/2 was the critical value, Ω represented the standard deviation, and n was the population size. Normal Gaussian distribution was applied through the Shapiro-Wilk goodness-of-fit test (z value > 1.0) to evaluate the normality of data distribution. Besides, repeated measures ANOVA followed by Bonferroni post-test was used to assess parametric distributions (RMSSD, HF and LF in absolute units). For non-parametric distributions, on the other hand, Friedman test followed by Dunn’s test was applied (SDNN, pNN50, LF and HF in normalized units and LF/HF ratio). The HRV indices were also compared in the three moments (rest with no music, classical baroque music, and heavy metal music). It should be mentioned that music exposure was randomized in this study. First, the subjects remained at seated rest for 10 minutes. Subsequently, the volunteers were exposed to heavy metal or classical music for five minutes. Five minutes after the first music ended, the individuals were exposed to the opposite music. P < 0.05 was considered as statistically significant. All the analyses were performed using GraphPad StatMate software, version 2.00 for Windows and GraphPad Software, San Diego California USA.

## 4. Results

**Figure 1.**
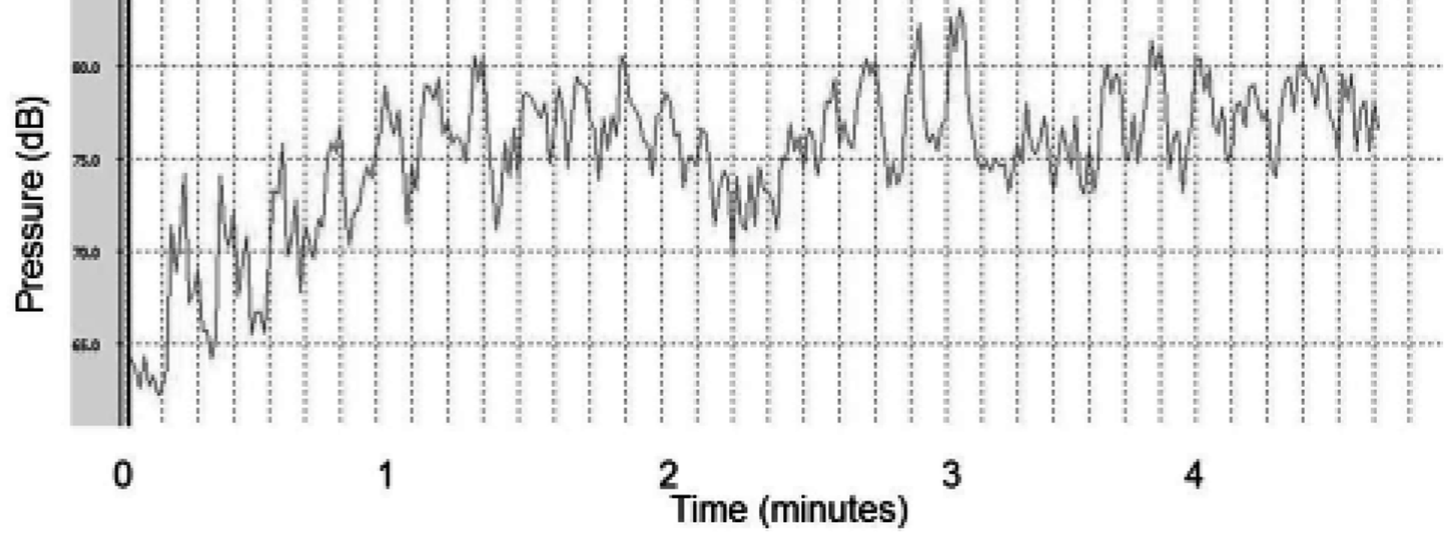
Equivalent Sound Level of Classical Musical Auditory Stimulation (dB: Decibel)

**Figure 2.**
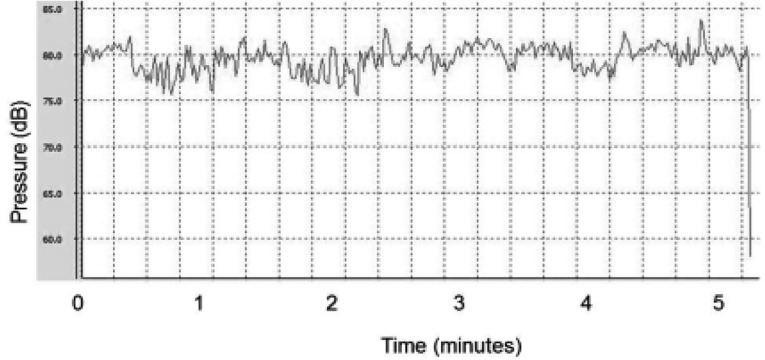
Equivalent Sound Level of Heavy Metal Musical Auditory Stimulation (dB: Decibel)

The measurements related to classical baroque music and the sound intensity during heavy metal music stimulation have been presented in Figures 1 and 2, respectively.

In addition, the data regarding baseline systolic (SAP) and diastolic arterial pressure (DAP), Heart Rate (HR), mean RR interval, age, height, body weight, and BMI have been shown in Table 2.

**Table 2.** The Volunteers’ Baseline Diastolic (DAP) and Systolic Arterial Pressure (SAP), Heart Rate (HR), Mean RR Interval, Weight, Height, and Body Mass Index (BMI)

Variable	Value ^*^
**Age (years)**	22.6 + 2
**Height (m)** ^**a**^	1.75 + 0.06
**Weight (kg)**	64 + 12
**BMI (kg/m ^2^)** ^**a**^	22.17 + 2
**HR (bpm)** ^**a**^	82 + 9
**Mean RR (ms)** ^**a**^	745.4 + 90
**SAP (mmHg)** ^**a**^	110 + 10
**DAP (mmHg)**	60 + 10

^*^ Mean + standard-deviation

^a^ Abbreviations: m, meters; ms, millisecond; kg, kilograms; bpm, beats per minute; mmHg, millimeters of mercury

With respect to the time-domain index, significant changes were observed during exposure to classical baroque and excitatory heavy metal musical auditory stimulation. According to the results, the SDNN index reduced during excitatory heavy metal music exposure compared to the control condition (P = 0.023). However, no significant changes were observed in the RMSSD (P = 0.900) and PNN50 indices (P = 0.900) during musical auditory stimulation with both music styles (Table 3).

**Table 3.** Mean and Standard Deviation of Time-Domain Indices before and during Musical Auditory Stimulation

Index	Control	Baroque Music	Heavy Metal Music
**SDNN (ms)** ^**a**^	51.6 + 15	52.1 + 12	44.5 + 11 ^*^
**RMSSD (ms)** ^**^a^**^	30 + 13	29.9 + 12	29 + 14
**pNN50 (%) ** ^**a**^	11.7 + 9	11.8 + 10	12 + 12

^*^ P < 0.05: Vs. Control

^a^ Abbreviations: SDNN, standard deviation of normal-to-normal R-R intervals; pNN50, the percentage of adjacent RR intervals with a difference of duration greater than 50 ms; RMSSD, root-mean square of differences between adjacent normal RR intervals in a time interval; ms, millisecond

The study results regarding the frequency-domain indices in response to musical auditory stimulation revealed more intense reactions in LF (ms^2^) (P = 0.010), HF (ms^2^) (P = 0.010), and LF/HF ratio (P = 0.020) indices. Moreover, the LF (ms^2^) index was significantly reduced during excitatory heavy metal and classical baroque musical auditory stimulation compared to the control condition (Table 4). Nevertheless, the HF (ms^2^) index was reduced only during heavy metal musical auditory stimulation. Furthermore, the LF (nu) (P = 0.048) and LF/HF ratio (P = 0.019) were decreased during baroque musical auditory stimulation. On the other hand, no significant changes were observed with respect to the HF (nu) (P = 0.250) (Table 4).

**Table 4.** Mean and Standard Deviation of Frequency-Domain Indices before and during Musical Auditory Stimulation

Index	Control	Baroque Music	Heavy Metal Music
LF (ms^2^) ^a^	1269 + 648	801 + 473 ^*^	839.5 + 444 ^*^
HF (ms^2^) ^a^	395.1 + 288	461.5 + 419	370 + 365 ^*^
LF (nu) ^a^	77.9 + 10	66.7 + 19 ^*^	72.7 + 18
HF (nu)	22.11 + 10	33.3 + 19	25.8 + 16
LF/HF ^a^	4.25 + 2.2	3.28 + 3.9 ^*^	4.39 + 3.7

^*^ P < 0.05: Vs. Control

^a^ Abbreviations: LF, low frequency; HF, high frequency; LF/HF, low frequency/high frequency ration; ms, milliseconds; nu, normalized units

## 5. Discussion

The present study aimed to investigate the acute effects of musical auditory stimulation with different styles on HRV in healthy males. The results showed that excitatory heavy metal music decreased SDNN, LF (ms^2^ and nu), and HF (ms^2^). The classical baroque style musical auditory stimulation, on the other hand, reduced the LF in absolute units and the LF/HF ratio. These results suggested that acute exposure to excitatory heavy metal music reduced the global HRV, affecting the sympathetic and parasympathetic components of HRV, while classical baroque music was involved in the sympathetic regulation of the heart. The novelty of our study is the use of earphones, since most of the studies in the literature exposed the subjects to music through stereo system (3, 4).

Considering time-domain indices analysis, no significant changes were observed in the RMSSD and pNN50 indices in response to acute exposure to heavy metal and classical baroque music styles. These indices represent the influence of the parasympathetic system on the heart (15). On the other hand, heavy metal music style decreased the SDNN index. The SDNN index corresponds to the sympathetic and parasympathetic components; however, this index does not provide information to distinguish whether changes in the autonomic cardiac regulation are due to increased sympathetic tone or vagal withdrawal (1). A previous study investigated the SDNN index in peritoneal dialysis patients. The study results indicated a significant reduction in this index in the patients who presented with residual renal function decline (16). The SDNN index has also been observed to be chronically influenced by music. Chuang et al. (4) exposed anthracycline-treated breast cancer patients to eight weekly music therapy sessions. The music used in that study was popular Taiwanese songs with pleasant, moderate rhythms and tempos. The researchers reported an increase in SDNN at the 8th week. However, this index returned to the pre-treatment value two weeks after musical therapy cessation (4). We believe that acute heavy metal music exposure caused opposite responses in our study because this music style presents intense rhythm and is more stirred.

Considering frequency-domain analysis, the study findings revealed that excitatory heavy metal music stimulation reduced the LF in absolute units. The LF index corresponds to both vagal and sympathetic influences on the heart, yet providing predominance of the sympathetic component (1). Similar to our study, Iwanaga et al. (17) investigated HRV responses to acute sedative and excitatory music (17). They also observed that the LF index reduced during excitatory music exposure compared to no music stimulation. The excitatory music used in the aforementioned study was Igor Stravinsky’s ‘‘Sacrificial Dance’’ from The Rite of Spring performed by the Detroit Symphony Orchestra, Antal Dorati conducting (Decca 400 084-2). This portion of the music is wild, rhythmic, and dynamic. It is performed violently with percussion and brass instruments. In the current study, heavy metal was used as an excitatory music. We believe that excitatory music acutely reduces the sympathetic tone on the heart. Nevertheless, furthers studies are necessary to be conducted on different styles of excitatory music to confirm this hypothesis.

One suggested mechanism that may explain the reduced global modulation of the heart during heavy metal music is the accelerating rhythm of the music. This auditory stimulation is suggested to cause changes in the cardiac autonomic regulation, eventually reducing the global variability of the heart rate (8).

The findings of the present study demonstrated that excitatory heavy metal musical auditory stimulation reduced the HF index in absolute units. The HF index indicates the action of the parasympathetic nervous system on the heart. It is also an indicator of the respiratory modulation influence on the heart (1). The power spectral analysis indices in absolute units undergo more influence of the endocrine regulation; i.e., renin-angiotensin system. However, this system can be measured only for a period of 20 minutes (1), ruling out the possibility of endocrine responses induced by heavy metal music. We expected a decrease in parasympathetic values in response to heavy metal music, since the literature has suggested that this style of music may cause exhaustion, restlessness, sleep disturbances, fatigue, stress, impairment of the immune system, hearing difficulties, and/or hearing loss (18). Heavy metal is considered to be ineffective or even harmful to human health. This music style induces violent behavior, dissatisfaction, and rage as well as an increase in both heart rate and blood pressure. Moreover, breastfeeding mothers should avoid this music because it was previously observed to have a negative influence on milk flow in pregnant women (18).

The present study results showed that the classical baroque music acutely influenced only the frequency-domain indices of HRV. The results indicated a decrease in the HF in absolute units, the LF in normalized units, and the LF/HF ratio during exposure to Pachelbel: Canon in D music. The literature has demonstrated that musical auditory stimulation presented reliable physiological responses. The subjects’ responses were related to faster breathing, were associated to tempo and were not influenced by musical preferences (19, 20). The chronic effects of relaxant musical auditory stimulation on cardiovascular system were described as beneficial by some authors (4, 21, 22). A previous investigation showed that sedative music stimulation acutely reduced the LF index and the LF/HF ratio, but increased the HF index in healthy male and female subjects (17). However, the acute effect of music on HRV has not been clearly stated in the literature. The music used in the above-mentioned study was the orchestral version of Erik Satie’s Gymnopedie No. 1 arranged by Claude Debussy and performed by the Academy of St. Martin-in-the-Fields, Sir Neville Marriner conducting (Philips 420 155-2). This music is soft, harmonious, beautiful, and slight. Based on the study by Iwanaga et al. and the present one, relaxant music acutely reduced the sympathetic modulation of the heart (17).

The music intensity used in our study ranged between 60 and 80 dB. Based on the research by Lee et al., auditory stimulation above 50 dB without interference (white noise) increased the sympathetic activity and arterial blood pressure in rats (23). The same researchers also found a strong correlation between the sympathetic-vagal balance through analysis of the LF/HF ratio and auditory stimulation intensity. In addition, subcortical processing centers and cortical centers were hypothesized to be involved in the hormonal and cardiovascular responses to long-term stress activation by environmental noises, even when the noise intensity was as low as 53 dB (24).

Up to now, some studies have investigated the mechanisms involved in physiological responses induced by musical auditory stimulation. A previous study indicated a direct sign that the feelings experienced when hearing music was related to the mesolimbic reward dopamine system (25). The feelings evoked by music were induced by tension, delay, resolution, prediction, surprise, expectations, and anticipation (26). In fact, a temporal dissociation was reported among distinct regions of the striatum while listening to enjoyable music (18).

The associate neurochemical, hemodynamic, and psychophysiological protocols used showed that peaks of ANS, reflecting the experience of the most intense emotional moments, were associated with dopamine release in the nucleus accumbens. Nucleus accumbens is involved in the euphoric element of psychostimulants, such as cocaine, and is highly interconnected with limbic regions that mediate emotional responses, such as the hippocampus, cingulate, amygdala, and ventromedial prefrontal cortex (26, 27).

Our investigation presents some points that should be raised. The present study was conducted on a small population; however, the number of the volunteers was higher than the number provided by the power test analysis and the statistical analyses provided significant differences for the investigated indices. Moreover, only healthy male subjects were evaluated in order to homogenize the study sample. Thus, the results should be generalized to female gender and different pathological conditions with due caution. Furthermore, respiratory rate is also influenced by the autonomic nervous system (28), but we did not measure the respiratory rate during musical auditory stimulation. Of course, our study aimed to focus on the cardiac autonomic regulation. In order to avoid the influence of positive emotions on the effects of music on HRV, we excluded the volunteers who enjoyed the music. As previously mentioned, emotional responses are induced by music (18) and emotion is associated with the autonomic nervous system (29). Nevertheless, we did not measure the volunteers’ emotional responses. Hence, further studies are recommended to investigate the respiratory and emotional responses to musical auditory stimulation and their interaction with cardiac autonomic regulation.

The findings of the present study showed that acute exposure to classical baroque music reduced the sympathetic modulation of the heart, while excitatory heavy metal music decreased the global variability of the heart rate. Moreover, the classical baroque music acutely increased HRV in healthy male subjects.
